# ZnO Nanocages Decorated with Au@AgAu Yolk–Shell Nanomaterials for SERS-Based Detection of Hyperuricemia

**DOI:** 10.1021/acsomega.3c10057

**Published:** 2024-03-28

**Authors:** Mei-Chin Lien, I-Hsiu Yeh, Sirimuvva Tadepalli, Keng-Ku Liu

**Affiliations:** † Department of Biomedical Engineering and Environmental Sciences, National Tsing Hua University, Hsinchu 300044, Taiwan; ‡ Department of Microbiology and Immunology, Stanford University School of Medicine, Stanford, California 94305, United States

## Abstract

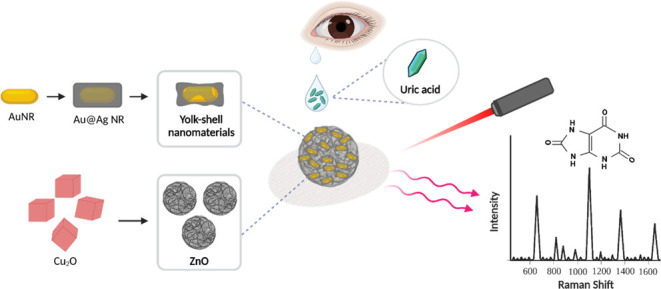

Surface-enhanced Raman scattering (SERS) is widely recognized as a highly sensitive technology for chemical detection and biological sensing. In SERS-based biomedical applications, developing highly efficient sensing platforms based on SERS plays a pivotal role in monitoring disease biomarker levels and facilitating the early detection of cancer biomarkers. Hyperuricemia, characterized by abnormally high concentrations of uric acid (UA) in the blood, was associated with a range of diseases, such as gouty arthritis, heart disease, and acute kidney injury. Recent reports have demonstrated the correlation between UA concentrations in blood and tears. In this work, we report the fabrication of SERS substrates utilizing ZnO nanocages and yolk–shell-structured plasmonic nanomaterials for the noninvasive detection of UA in tears. This innovative SERS substrate enables noninvasive and sensitive detection of UA to prevent hyperuricemia-related diseases.

## Introduction

Surface-enhanced Raman scattering (SERS), a highly sensitive and nondestructive analytical technology, is widely recognized as a powerful spectroscopic approach for chemical detection and biological sensing.^1−3^ Recent years have seen a surge in research efforts focused on fabricating SERS substrates with high sensing efficiency, enabling trace detection of analytes and providing the fingerprint-level resolution of single molecules.^4−10^ In SERS-based biomedical applications, developing highly efficient sensing platforms based on SERS plays a pivotal role in monitoring disease biomarker levels and facilitating early detection of cancer biomarkers.^11−14^ Consequently, a growing number of studies are exploring the application of SERS as a label-free diagnostic tool for chemical and biological sensing.^12,15−17^

Due to the electron transfer between the adsorbed molecules and the semiconducting metal oxide nanostructures under laser excitation, various semiconducting metal oxide nanostructures have been reported to provide high Raman enhancement.^18−20^ The resulting significant Raman signals make metal oxide semiconductors excellent candidates for SERS-based sensing.^21^ In addition to semiconducting materials, noble metal nanomaterials have garnered considerable research interest as SERS-active materials, thanks to the surface plasmon effect resulting from the electromagnetic fields on the metal surface upon excitation.^22,23^ Hollow and porous plasmonic nanomaterials are relatively novel nanostructures owing to their unique structures, high surface-to-volume ratio, interior vacancies, and tunable localized surface plasmon resonance (LSPR) properties.^24−27^ Yolk–shell-structured plasmonic nanomaterials have been reported to demonstrate superb optical properties because of the high density of electromagnetic fields.^28−30^ With increasing focus on research dedicated to advancing highly efficient transducers, yolk–shell nanomaterials have emerged as a promising avenue for achieving optimal sensing capabilities. Moreover, the combination of semiconducting metal oxide and noble metal nanomaterials (e.g., ZnO/Au nanoarray, ZnO/Ag nanoparticles, Au-coated ZnO nanorods) has been demonstrated for SERS-based detection of microplastics, volatile organic compounds, and chemicals.^31−33^

Hyperuricemia, characterized by abnormally high levels of uric acid (UA) in the blood, has been linked to various diseases such as gouty arthritis, heart disease, and acute kidney injury.^34,35^ Individuals with UA concentration in serum surpassing 480 μM have been identified with hyperuricemia-related diseases.^36,37^ The early and sensitive detection of disease biomarkers is vital for clinical diagnosis and point-of-care (POC) testing. Noninvasive, inexpensive, and user-friendly sensing platforms are also essential for POC diagnosis. Tears, encompassing a diverse repertoire of biomolecules such as peptides, proteins, lipids, and metabolites, hold great promise as an accessible source of body fluids for various medical diagnostics in POC.^11,38^ According to a recent report, a robust correlation has been identified between the concentrations of UA present in both the blood and tears.^11^ Normally, the concentrations of UA in human tears are found to be ranging from 25 to 150 μM, and the average level is 68 ± 46 μM.^11^ Hence, accurate and sensitive detection of UA in tears holds promising potential as a noninvasive method for POC diagnosis.

Herein, we report the fabrication of SERS substrates using the ZnO nanocages and the yolk–shell nanomaterials for the noninvasive detection of UA in tears. This sensing method enables noninvasive and sensitive detection of UA to prevent hyperuricemia-related diseases.

## Results and Discussion

**Figure 1:**
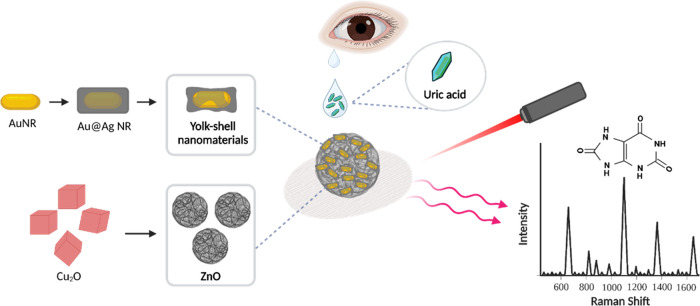
Schematic illustration depicting the concept of the preparation of ZnO nanocages decorated with Au@AgAu yolk–shell nanomaterials for SERS-based diagnosis of hyperuricemia.

**Figure 2:**
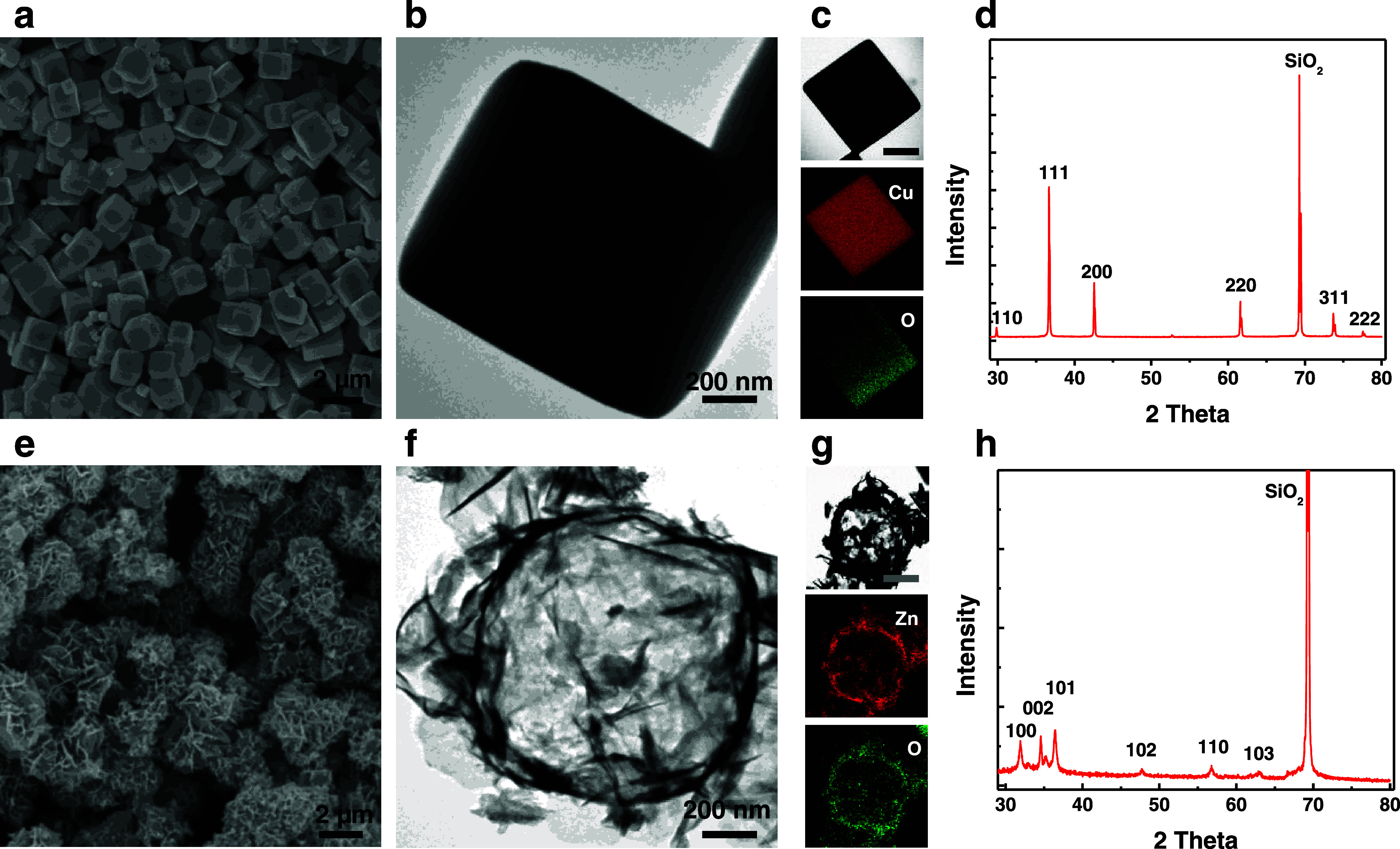
(a) SEM and (b) TEM images of Cu_2_O nanocubes. (c) HAADF STEM image and the elemental mapping of the Cu_2_O nanocube. The scale bar is 500 nm. (d) XRD spectrum of Cu_2_O nanocubes. (e) SEM and (f) TEM images of ZnO nanocages. (g) HAADF STEM image and the elemental mapping of the ZnO nanocage. The scale bar is 500 nm. (h) XRD spectrum of ZnO nanocages. SiO_2_ peaks in (d, h) resulted from the SiO_2_/Si substrate.

Figure 1 is a conceptual illustration showing the SERS substrates based on the ZnO nanocages and the yolk–shell nanomaterials for the sensitive detection of UA in tears. ZnO nanomaterials have been reported to be promising candidates for preparing SERS substrates due to their facile synthesis, optical stability, and tunable morphology.^32,39^ A template-assisted route achieved by a previously reported method with slight modification was employed for the synthesis of ZnO nanocages.^39−41^ Briefly, the Cu_2_O nanocubes served as the templates, which were prepared by the heating of the aqueous solution of CuCl_2_·2H_2_O at 55 °C, followed by the addition of the aqueous solution of NaOH and the titration of the aqueous solution of ascorbic acid (please see the Experimental Section for details). The edge length of the as-synthesized Cu_2_O nanocubes was measured to be 0.97 ± 0.14 μm (Figures 2a and S1). The high-angle annular dark-field (HAADF) STEM-EDX elemental mapping images reveal the distribution of copper and oxygen (Figure 2c). In Figure 2d, the X-ray diffraction (XRD) pattern showed the related peak positions corresponding to the plane values of (110), (111), (200), (220), (311), and (222), which were in agreement with the structure of the Cu_2_O.^42^ X-ray photoelectron spectroscopy (XPS) was used to investigate the chemical composition of the Cu_2_O nanocubes (Figure S2). The XPS spectrum of Cu_2_O nanocubes revealed the presence of copper and oxygen elements. The as-prepared Cu_2_O nanocubes were then dispersed in ethanol/water (in a ratio of 1/1) in the presence of zinc chloride (ZnCl_2_) and poly(vinylpyrrolidone) (PVP). An aqueous solution of sodium thiosulfate (Na_2_S_2_O_3_) was injected into the solution and kept at room temperature until the suspension changed from red to white. The obtained white precipitates, Zn(OH)_2_ nanospheres, were collected and washed with nanopure water and ethanol. The edge length of the as-synthesized Zn(OH)_2_ nanospheres was measured to be 2.52 ± 0.17 μm (Figure S3). The dehydration reaction was performed to convert the Zn(OH)_2_ nanospheres into ZnO nanocages at 200 °C. The edge length of the as-synthesized ZnO nanocages was measured to be 2.62 ± 0.15 μm (Figure 2e,f). The XPS spectra of ZnO nanocages revealed the presence of zinc and oxygen elements (Figure S4). The distribution of zinc and oxygen in the ZnO nanocages is shown in the HAADF STEM-EDX elemental mapping images (Figure 2g). XRD measurements revealed the plane values of (100), (002), (101), (102), (110), and (103), which further confirmed the formation of wurtzite ZnO nanostructures (Figure 2h). The surface areas of the Cu_2_O nanocubes and ZnO nanocages were determined by analyzing N_2_ adsorption–desorption curves using the Brunauer–Emmett–Teller (BET) method. The resulting measurements indicated surface areas of 1.47 m^2^/g for Cu_2_O nanocubes and 30.75 m^2^/g for ZnO nanocages (Table S1).

**Figure 3:**
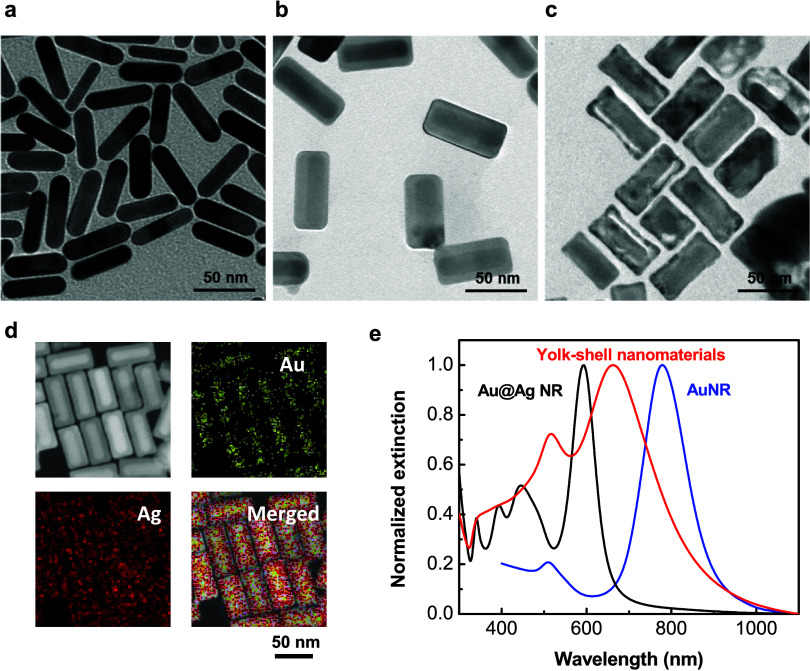
TEM images of (a) AuNRs, (b) Au@Ag NRs, and (c) yolk–shell nanomaterials. (d) HAADF STEM and the elemental mapping of Au, Ag, and merged images. (e) Normalized extinction spectra of AuNRs, Au@Ag NRs, and yolk–shell nanomaterials.

Yolk–shell nanomaterials were synthesized using a procedure in our recent report.^30^ The procedure started with synthesizing gold nanorods (AuNRs) that served as the cores in yolk–shell nanomaterials.^43,44^ The dimensions of the AuNRs were determined to be 53.5 ± 3.6 nm in length and 14.5 ± 1.7 nm in width (Figures 3a, S5a, and S6a,d). A deposition technique involving the utilization of silver nitrate as the silver precursor, ascorbic acid as the reducing agent, and hexadecyltrimethylammonium chloride as the surfactant was employed to facilitate the growth of a silver layer on the surface of the AuNRs. Transmission electron microscopy (TEM) and scanning electron microscopy (SEM) images reveal the uniform growth of silver on the surface of AuNRs, and the length and diameter of the Au@Ag NRs were measuring 58.8 ± 3.4 and 30.7 ± 2.5 nm, respectively (Figures 3b, S5b, and S6b,e). The HAADF STEM image, elemental mapping, and energy-dispersive X-ray spectroscopy (EDS) spectrum revealed the bimetallic structure of the Au@Ag (Figure S7). The galvanic replacement was used to transform Au@Ag NRs into Au@Au/Ag yolk–shell nanomaterials (Figures 3c and S5c,d). The size of yolk–shell nanomaterials was 59.9 ± 3.7 and 30.2 ± 2.7 nm (Figure S6c,f). The distribution of gold and silver in the yolk–shell nanomaterials is shown in the HAADF STEM image and corresponding EDX elemental mapping images (Figure 3d). The extinction spectrum revealed that the AuNRs displayed distinct plasmon resonance peaks at 510 and 779 nm (Figure 3e). After the silver coating, the main peak of the Au@Ag NRs in the LSPR spectrum was measured to be 592 nm. By titration of the suspension of Au@Ag NRs with an aqueous HAuCl_4_ solution, the dominant band of the resultant yolk–shell nanomaterials in the LSPR spectrum was measured to be around 662 nm.

**Figure 4:**
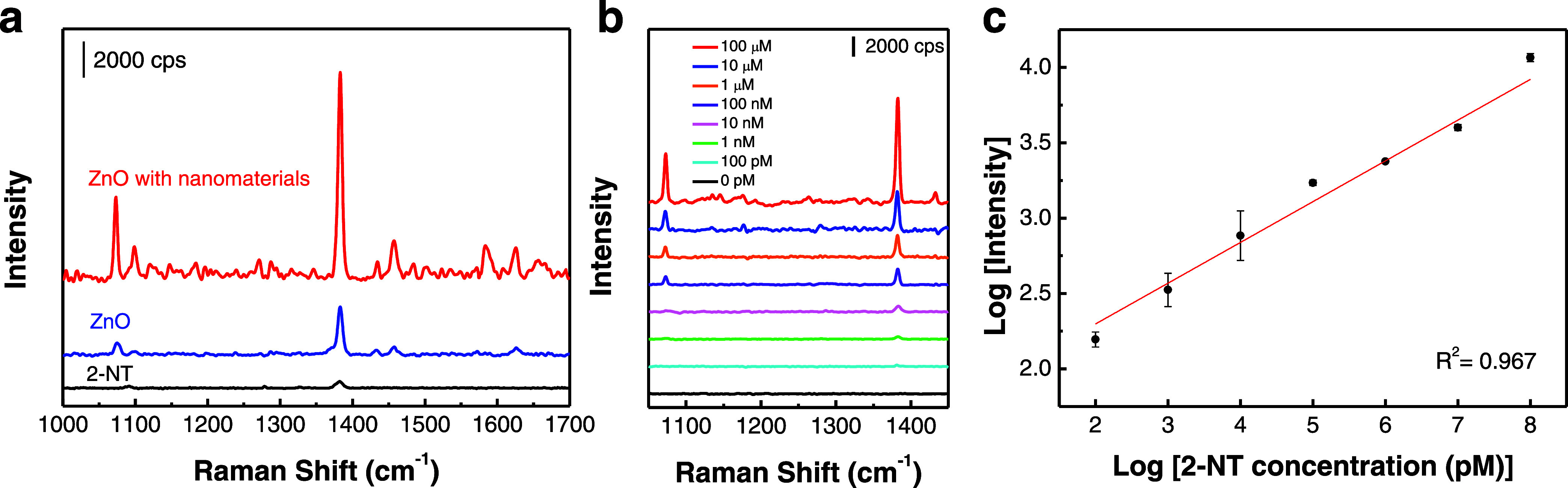
(a) Raman spectra of 2-NT and 2-NT-exposed ZnO nanocages with and without the decoration of yolk–shell nanomaterials. (b) Raman spectra of plasmonic biochips at a range of concentrations of 2-NT. (c) Raman intensity of the 1383 cm^–1^ Raman band collected from the plasmonic biochips at a range of concentrations of 2-NT. *n* = 3.

To prepare the SERS substrates, yolk–shell nanomaterial-decorated ZnO nanocages were immobilized onto the filter paper to prepare the SERS substrates (Figure S8). To evaluate the SERS activity of the SERS substrates, the substrates immobilized with ZnO nanocages, both with and without the presence of yolk–shell nanomaterials, were subjected to exposure to the Raman-active molecule 2-naphthalenethiol (2-NT). A Raman spectrometer with a 632.8 nm-wavelength excitation source was used for the measurement. Raman spectra obtained from the SERS substrates exposed to the Raman reporter revealed the bands at 1073 and 1383 cm^–1^, corresponding to the vibration modes of C–H bending and ring stretching in 2-NT, respectively (Figure 4a).^26^ The Raman intensity obtained from the ZnO nanocages exposed to 2-NT exhibited a higher value in comparison to the intensity obtained from 2-NT alone, indicating an augmentation in the Raman signal attributed to the ZnO nanocages.^39^ Moreover, the SERS intensity generated by the ZnO nanocages in the presence of yolk–shell nanomaterials exhibited a higher magnitude compared to that where nanomaterials were absent. This can be attributed to the synergistic enhancement effect from the plasmonic nanomaterial/ZnO hybrid platform that boosts the Raman enhancement.^45^ The enhancement factor (EF) of yolk–shell nanomaterial-decorated ZnO nanocages is estimated to be 7.6 × 10^6^ (see the Supporting Information for details). Figure 4b displays the SERS spectra acquired from the SERS substrates incorporating yolk–shell nanomaterial-decorated ZnO nanocages with varying concentrations of 2-NT. Notably, the SERS intensity exhibited a noticeable augmentation corresponding to the escalating concentration of 2-NT (Figure 4c).

**Figure 5:**
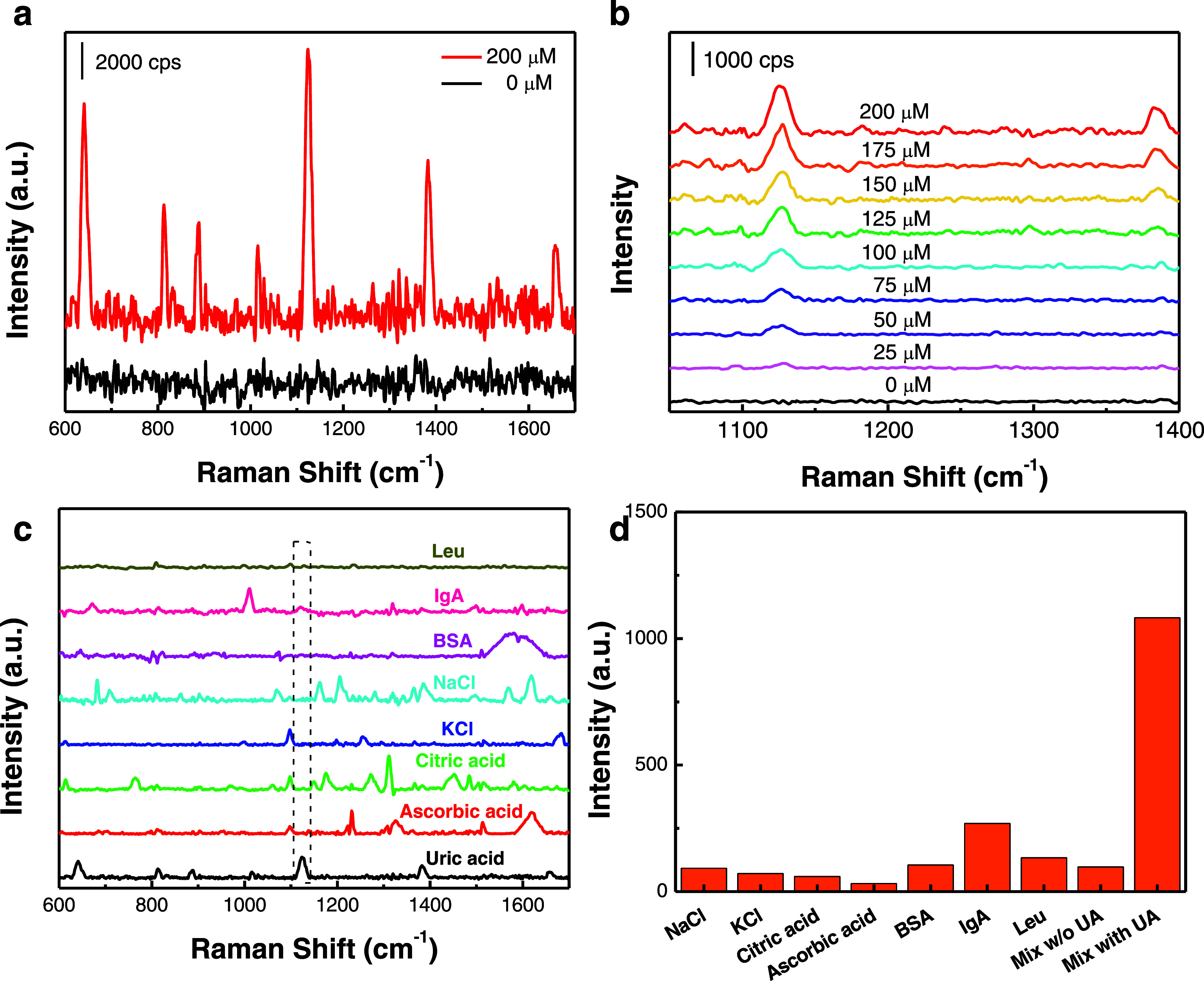
(a) Raman spectra of plasmonic biochips w/and w/o UA. (b) Raman spectra of plasmonic biochips with different concentrations of UA in artificial tears. (c) Raman spectra of plasmonic biochips with uric acid, ascorbic acid, citric acid, KCl, NaCl, BSA, IgA, and Leu exposure. (d) Raman intensity at the 1127 cm^–1^ band.

Next, we turn our attention to the trace detection capability of the plasmonic biochips, which utilize yolk–shell nanomaterial-decorated ZnO nanocages in the context of detecting UA. The detection capability of the plasmonic biochips was examined through exposure to various concentrations of UA in artificial tears (Figure 5a,b). The Raman spectra collected from the plasmonic biochips exposed to UA exhibited distinct Raman bands at 640, 813, 888, 1015, 1127, 1383, and 1658 cm^–1^, consistent with the Raman spectrum of UA powder and the previous findings as reported (Figure S9).^46^ The plot depicting the relationship between the UA concentration and the Raman band at 1127 cm^–1^ revealed a notable increase in Raman intensity as the UA concentration increased (Figure S10). The detection limit of UA was 25 μM, which is less than the average level of uric acid found in tears (Figure S11).^11^ Selectivity is another crucial factor that needs to be carefully investigated in the design and fabrication of biosensing platforms in clinical diagnosis and POC settings. To evaluate the selectivity of the plasmonic biochips for detecting UA, interfering experiments were carried out by testing the interfering molecules such as NaCl, KCl, citric acid, ascorbic acid, and BSA at a concentration of 200 μM and native human IgA and leucine enkephalin (Leu) at 1 μM. The testing spectra demonstrated that the interferences exhibited a minimal or undetectable Raman peak at 1127 cm^–1^ (Figure 5c,d). Conversely, with UA in the artificial tears, a distinct Raman peak at 1127 cm^–1^ was observed, thus verifying the discrimination capability of UA in the artificial tears. Furthermore, we conducted additional investigations to assess the stability of the SERS substrates in detecting UA. UA sensing experiments were carried out over 4 weeks to investigate the stability of the SERS substrates. Remarkably, the SERS intensities demonstrated consistency throughout tests, thereby pointing to the exceptional stability of the SERS substrates (Figures S12 and S13). Moreover, Raman intensities from 30 different regions across the yolk–shell nanomaterial-decorated ZnO nanocages with uric acid (200 μM) exposure show the reproducibility of the SERS substrate (Figure S14F). Traditional diagnosis of hyperuricemia typically involves invasive blood collection, specialized instruments, and skilled hospital operators to measure the blood UA level.^47^ In this study, the developed plasmonic biochips demonstrated the ability to sensitively, selectively, and noninvasively detect UA in artificial tears at concentrations within the physiological range. The achieved limit of detection for UA is lower than the average physiological level of UA in tears, indicating that the plasmonic biochips fabricated using the ZnO nanocages decorated with yolk–shell nanomaterials hold significant potential for sensitive sensing applications.

## Conclusions

In summary, this study presents the successful synthesis of ZnO nanocages and the utilization of yolk–shell nanomaterials to create a hybrid structure of yolk–shell nanomaterial-decorated ZnO nanocages, enabling SERS sensing of UA. The large Raman enhancement originated from the electromagnetic enhancement effect of yolk–shell nanomaterials, and the chemical enhancement effect of ZnO nanocages makes these hybrid plasmonic biochips promising for biosensing. The plasmonic biochips demonstrated remarkable sensing capabilities, effectively achieving a detection limit of 25 μM UA in artificial tears. Consequently, these plasmonic biochips are promising for highly sensitive and noninvasive diagnosis of hyperuricemia-related diseases in the POC setting.

## Experimental Section

### Materials

Copper(II) chloride dihydrate (CuCl_2_·2H_2_O), sodium hydroxide (NaOH), ascorbic acid, zinc chloride (ZnCl_2_), ethanol (≥99.8%), poly(vinylpyrrolidone) (K30), gold chloride trihydrate (HAuCl_4_·3H_2_O), hexadecyltrimethylammonium bromide (CTAB), sodium borohydride (NaBH_4_), silver nitrate (AgNO_3_), 2-naphthalenethiol (2-NT), uric acid (UA), sodium chloride (NaCl), potassium chloride (KCl), citric acid, and bovine serum albumin (BSA) were purchased from Sigma-Aldrich. Sodium thiosulfate pentahydrate (Na_2_S_2_O_3_·5H_2_O) was purchased from Honeywell Fluka. Hexadecyltrimethylammonium chloride (CTAC) was obtained from Tokyo Chemical Industry. Native human IgA was purchased from abcam. Leucine enkephalin was purchased from MedChemExpress. All chemicals were used as received without further purification.

### Synthesis of Cu_2_O Nanocubes

Cu_2_O nanocubes were prepared following a previously reported method.^40^ Briefly, 200 mL of the aqueous solution of CuCl_2_·2H_2_O (10 mM) was heated at 55 °C, and the color of the solution became light blue. 20 mL of the aqueous solution of NaOH (2M) was added into the solution under stirring (around 200–250 rpm), and the color of the solution gradually became dark brown gradually. After 30 min of stirring at 55 °C, 20 mL of the aqueous solution of ascorbic acid (0.6 M) was injected into the solution at a rate of 1 mL/min. The reaction was kept at 55 °C for 3 h. The precipitate was collected and washed with nanopure water and ethanol two times and then dried at 60 °C.

### Synthesis of Zn(OH)_2_ Nanospheres

Zn(OH)_2_ nanospheres were prepared based on a previous report with a slight modification.^39^ 0.1 g of Cu_2_O powders and 0.02 g of ZnCl_2_ were added into 10 mL of the ethanol/water (1/1) in the presence of 3.33 g of PVP. The mixture was sonicated for 10 min, and the color of the solution was red. 45 mL of Na_2_S_2_O_3_ aqueous solution (1M) was injected into the solution at a rate of 1 mL/min. The reaction mixture was kept at room temperature for 3 h until the color of the suspension changed from red to white. The white precipitate was collected and washed with nanopure water and ethanol two times and then dried at 60 °C.

### Synthesis of ZnO Nanocages

ZnO nanocages were prepared by heating Zn(OH)_2_ nanospheres at 200 °C for 30 min. The resulting product had a light-brown color and was ZnO nanocages.

### Preparation of Yolk–Shell Nanomaterials on ZnO Nanocages

ZnO nanocages (1.5 mg, suspended in ethanol) were placed onto a filter paper with a diameter of 0.6 cm, which was positioned in a well of a 96-well plate and left to dry naturally in the air. Next, 175 μL of yolk–shell nanomaterial suspension, with an extinction of approximately 2.0, was added to the surface of the filter paper in the well. The mixture was then left to dry naturally in the air.

### Characterization

UV–vis–NIR spectra were obtained by using a Shimadzu UV-1900 spectrophotometer. SEM images were obtained by using a JEOL JSM-7610F field emission instrument. TEM images were obtained by using a JEOL JEM-2100 field emission TEM. The STEM images were captured with a high-angle annular dark-field (HAADF) detector. Energy-dispersive X-ray spectroscopy (EDS) is affiliated with TEM. The X-ray diffraction (XRD) patterns of the samples were obtained using an X-ray powder diffractometer (Rigaku TTRAX III). XPS analysis was obtained using a high-resolution electron spectrometer (ULVAC-PHI).

### Spectroscopy Measurements

SERS measurements were obtained by using a Horiba Raman spectrometer (LABRAM HR 800 UV) mounted on a microscope equipped with a 50× objective and 632.8 nm-wavelength laser.

## Supporting Information Available

Synthesis of plasmonic nanomaterials, SEM image, histogram of edge length, XPS spectra of Cu_2_O microcubes, SEM image of Zn(OH)_2_ microspheres, XPS spectra of ZnO, BET surface areas of Cu_2_O and ZnO, SEM images of AuNRs, Au@Ag NRs, and yolk–shell nanomaterials, Raman spectrum of UA powder, stability and reproducibility test, and enhancement factor (PDF)
