# Supplementary material for: ZnO Nanocages Decorated with Au@AgAu Yolk–Shell Nanomaterials for SERS-Based Detection of Hyperuricemia
Source: ACS Omega. 2024 Mar 28;9(14):16160–7. doi: 10.1021/acsomega.3c10057 (PMC11007725; doi:10.1021/acsomega.3c10057)
Supplement: Supplementary file 1 — ao3c10057_si_001.pdf [file ao3c10057_si_001.pdf]

Supporting Information

ZnO Nanocages Decorated with Au@AgAu Yolk-Shell Nanomaterials for SERS-Based Detection of Hyperuricemia

Mei-Chin Lien[†], I-Hsiu Yeh[†], Sirimuvva Tadepalli [‡], and Keng-Ku Liu[†*]

[†] Department of Biomedical Engineering and Environmental Sciences, National Tsing Hua University, Hsinchu 300044, Taiwan

[‡.] Department of Microbiology and Immunology, Stanford University School of Medicine, Stanford, CA 94305, USA

*To whom correspondence should be addressed: kkliu@mx.nthu.edu.tw

Synthesis of Au Nanorods

Au nanorods were synthesized by using a seed-mediated method.[1, 2] Seed solution was synthesized by adding 0.6 ml of an ice-cold $NaBH_4$ (10 mM) solution into the solution containing 0.25 ml of $HAuCl_4$ (10 mM) and 9.75 ml of CTAB (0.1 M) under vigorous stirring at room temperature. The color of the seed solution changed from yellow to brown. The growth solution was prepared by mixing 5 ml of $HAuCl_4$ (10 mM), 95 ml of CTAB (0.1 M), 1 ml of $AgNO_3$ (10 mM), and 0.55 ml of ascorbic acid (0.1 M), consecutively. The solution was homogenized by gentle shaking. To the colorless solution, 0.12 ml of freshly prepared seed solution was added and kept undisturbed in the dark for 14 h. Before use, the AuNR solution was centrifuged twice at 8000 rpm for 10 min to remove excess CTAB and re-dispersed in nanopure water.

Synthesis of Au@Ag NRs

4 ml of twice-centrifuged AuNR and 8 ml of CTAC (20 mM) were mixed at 60 °C under stirring for 20 min. 1.6 ml of $AgNO_3$ (2 mM), 2 ml of CTAC (20 mM), and 0.8 ml of ascorbic acid (0.1M) were added under stirring at 60 °C for 4 h. The Au@Ag NRs solution was centrifuged at 8,000 rpm for 10 min and re-dispersed in 50 mM CTAC solution.

Synthesis of Yolk-shell Nanomaterials

Yolk-shell nanomaterials were synthesized by transforming the Ag shell of Au@Ag NRs into the porous shell of Au/Ag via a galvanic replacement reaction. The as-synthesized Au@Ag NRs were centrifuged and re-dispersed in CTAC solution (20 mM). $HAuCl_4$ aqueous solution (0.5 mM) was injected into the Au@Ag NRs solution at a rate of 0.5 ml/min under magnetic stirring until the desired LSPR wavelength was achieved.

a b
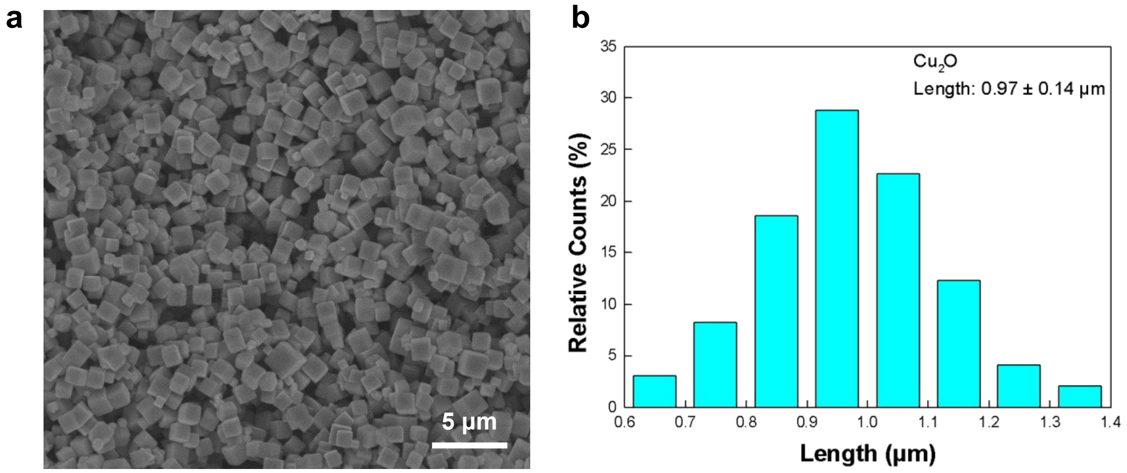

Figure S1. (a) SEM image of Cu_2O nanocubes. (b) Histogram of the edge length of Cu_2O nanocubes.

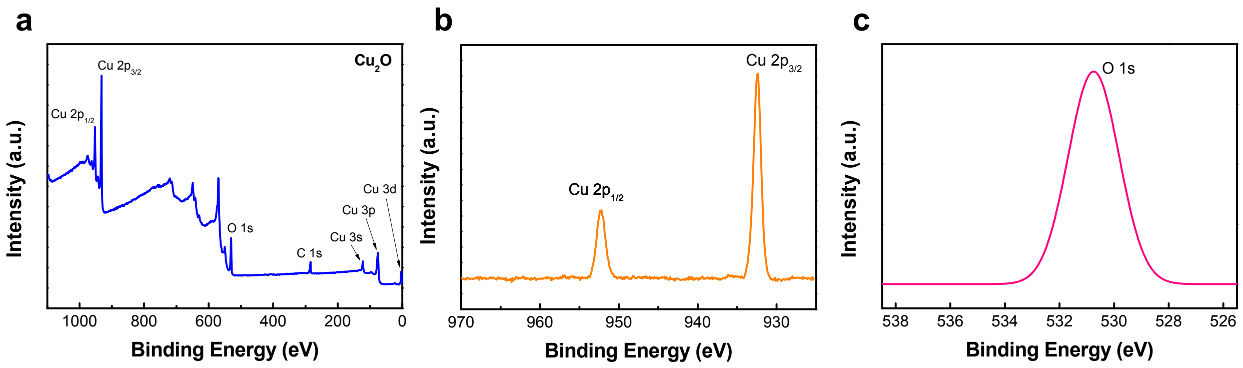

Figure S2. (a) XPS survey scan of Cu_2O and the binding energy for (b) copper (Cu $2p_{1/2}$ and Cu $2p_{3/2}$) and (c) oxygen (O 1s).

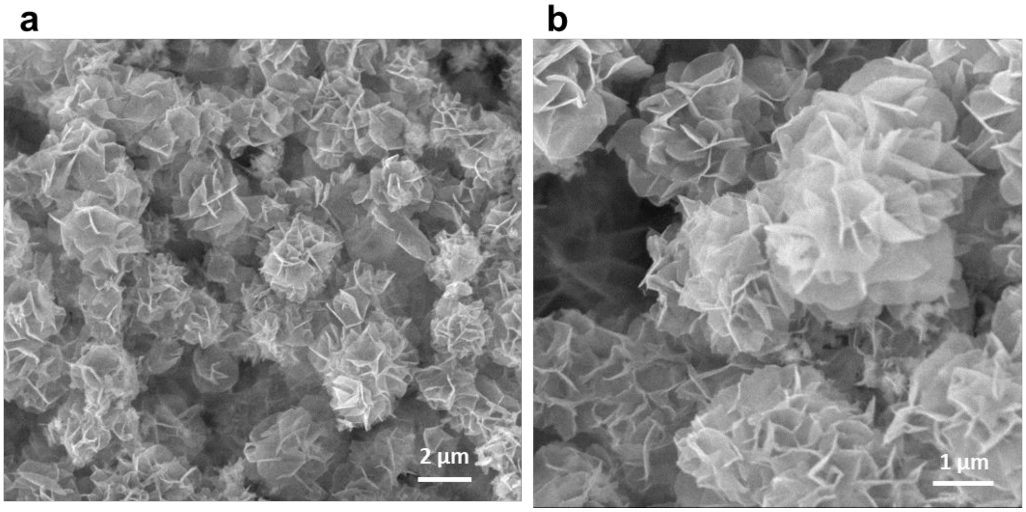

Figure S3. SEM image of $Zn(OH)_2$ nanospheres with relatively (a) low and (b) high magnifications.

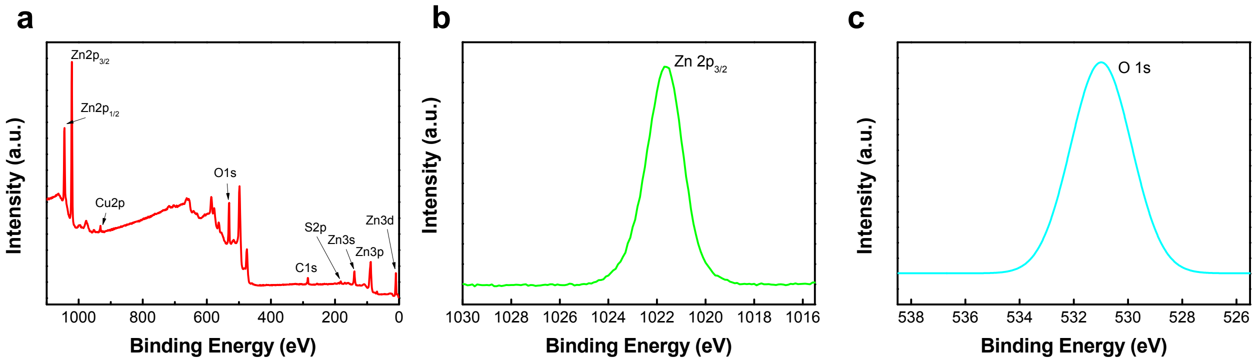

Figure S4. (a) XPS survey scan of ZnO and the binding energy for (b) zinc (Zn $2p_{3/2}$) and (c) oxygen (O 1s).

Table S1 Brunauer-Emmett-Teller (BET) surface areas of the Cu_2O nanocubes and ZnO nanospheres.

**Sample**	**BET (m^2/g)**
Cu_2O	1.47
ZnO	30.75

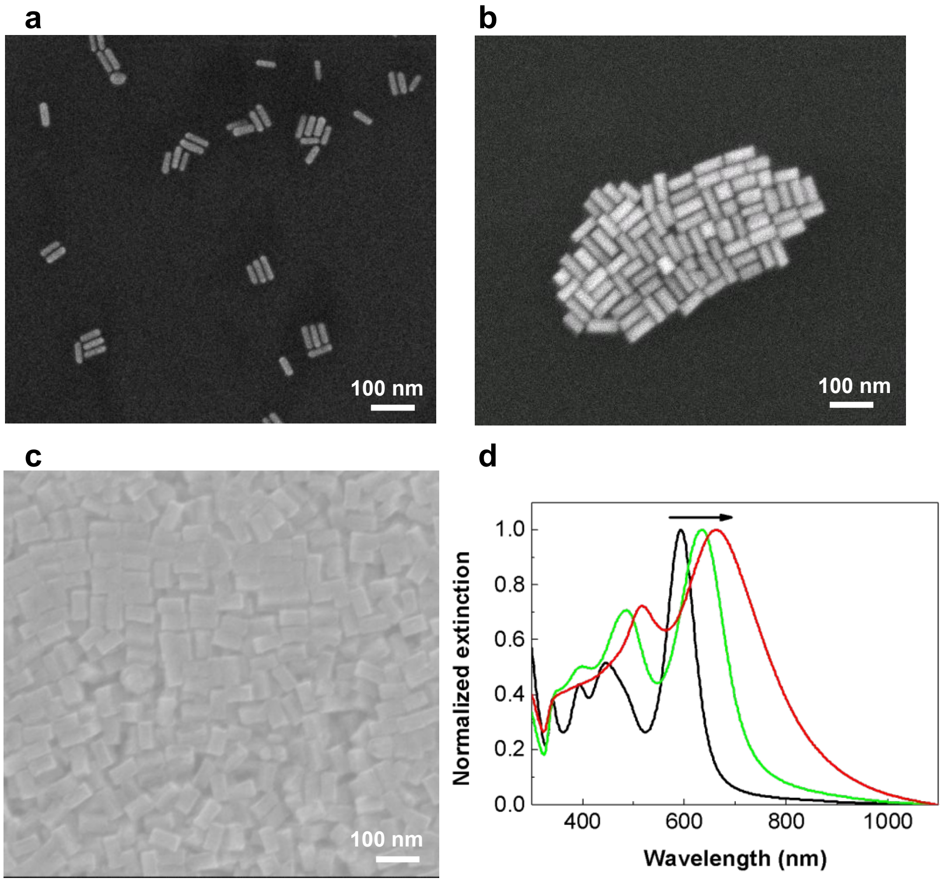

Figure S5. SEM images of (a) AuNRs, (b) Au@Ag NRs, and (c) yolk-shell nanomaterials. (d) Representative UV-Vis spectra of Au@Ag NRs upon addition of different volume of aqueous of $HAuCl_4$ solution during the galvanic replacement reaction to form the yolk-shell nanomaterials.

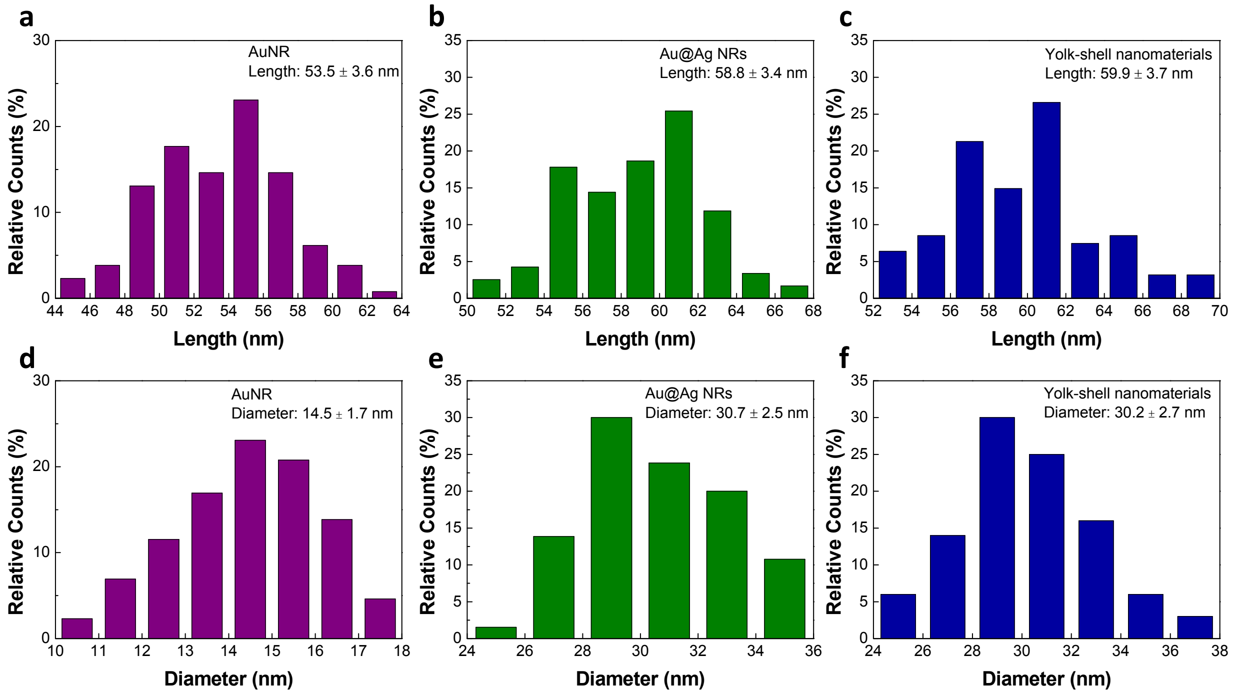

Figure S6. The histogram of the size distribution of the AuNRs, Au@Ag NRs, and yolk-shell nanomaterials as measured from TEM images. (a) Length of AuNRs. (b) Length of Au@Ag NRs. (c) Length of yolk-shell nanomaterials. (d) Diameter of AuNRs. (e) Diameter of Au@Ag NRs. (f) Diameter of yolk-shell nanomaterials. The number of particles measured to arrive at the relative counts for particle sizes are 130, 130, and 100 for AuNRs, Au@Ag NRs, and yolk-shell nanomaterials.

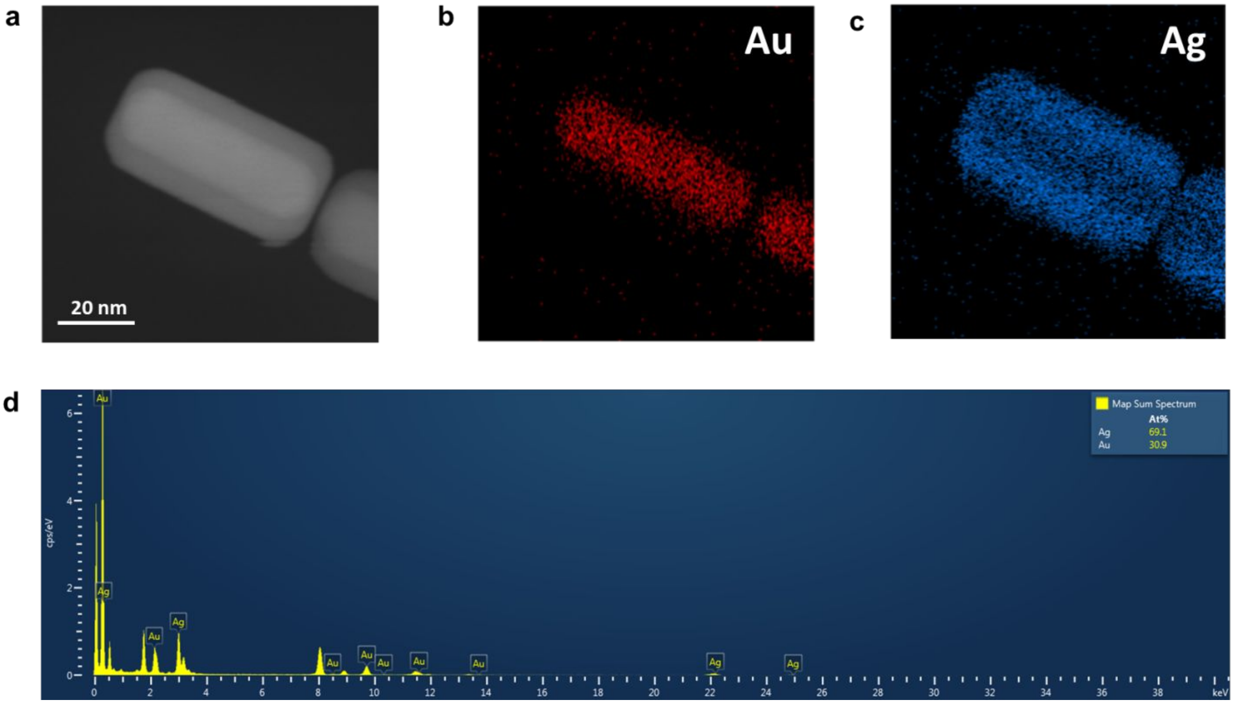

Figure S7. (a) HAADF STEM image of Au@Ag NR and the elemental mapping of (b) Au and (c) Ag. (d) EDS spectrum of Au@Ag NRs.

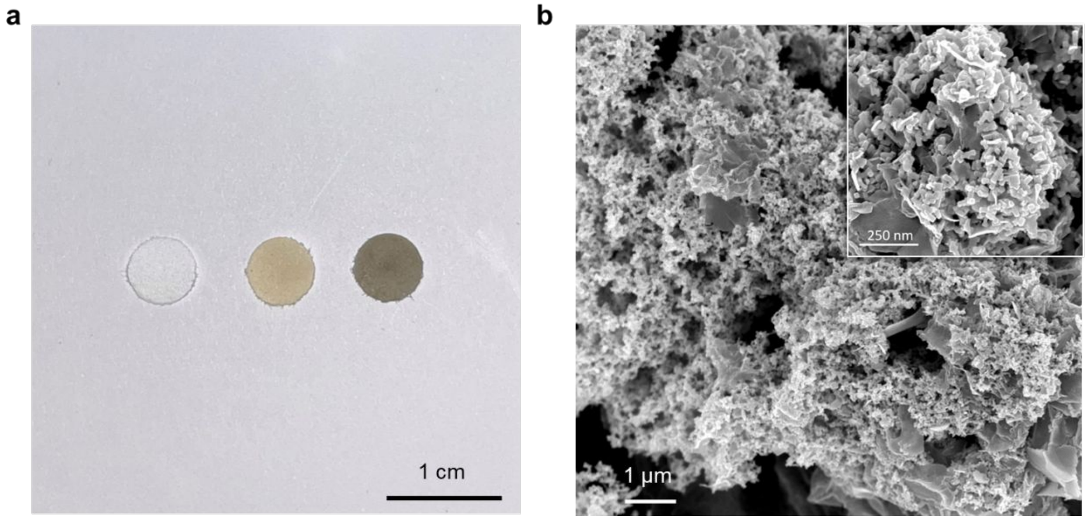

Figure S8. (a) Optical image shows the filter paper (left), ZnO nanocages on filter paper (middle), and yolk-shell nanomaterials-decorated ZnO nanocages on filter paper. (b) SEM images of yolk-shell nanomaterials-decorated ZnO nanocages with relatively low and high (inset) magnification.

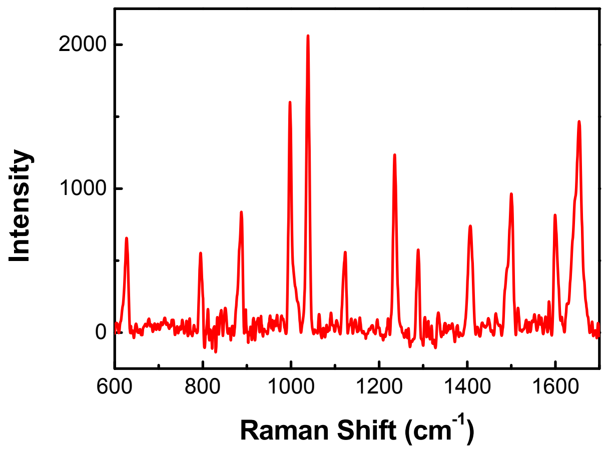

Figure S9. Raman spectrum of UA powder.

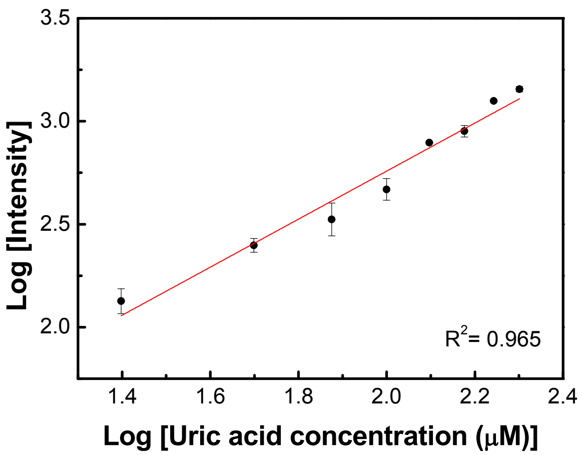

Figure S10. Plot showing the Raman intensity of the 1127 cm^{-1} Raman band obtained from the yolk-shell nanomaterials-decorated ZnO nanocages at various concentrations of UA in artificial tears. n=3.

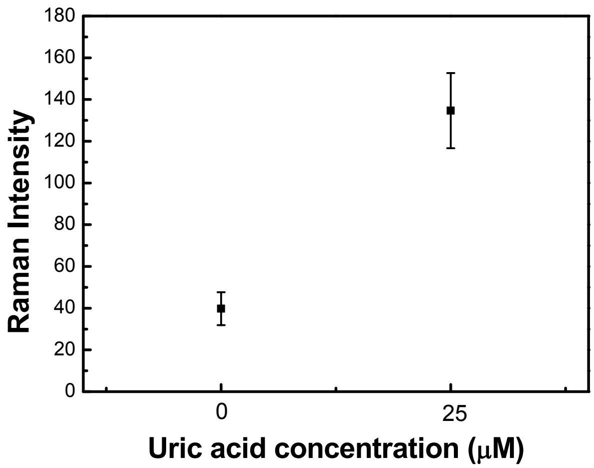

Figure S11. Raman intensity of the 1127 cm^{-1} Raman band obtained from the yolk-shell nanomaterials-decorated ZnO nanocages exposed to the blank sample and UA at the concentration of 25 μM in artificial tears. n=3.

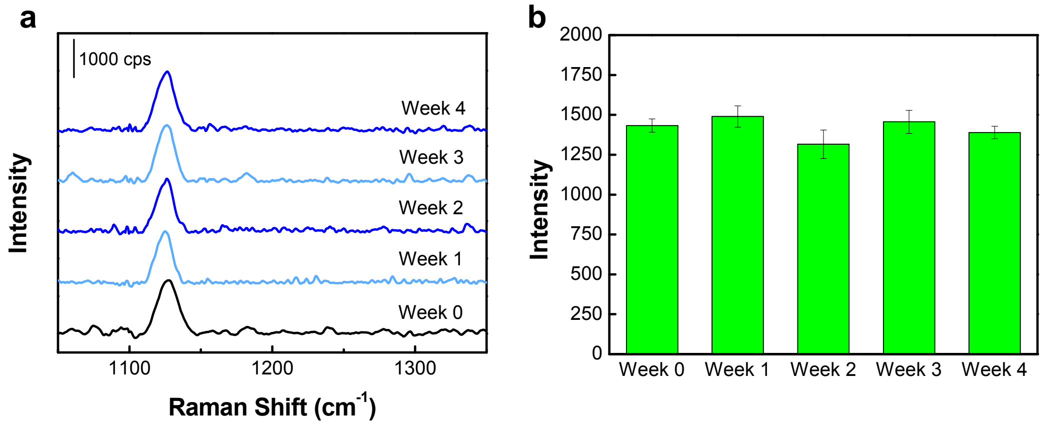

Figure S12. (a) Raman spectra obtained from the yolk-shell nanomaterials-decorated ZnO nanocages with uric acid exposure at various time points. (b) The histogram of the Raman intensity at the 1127 cm^{-1} Raman band in (a). n=3.

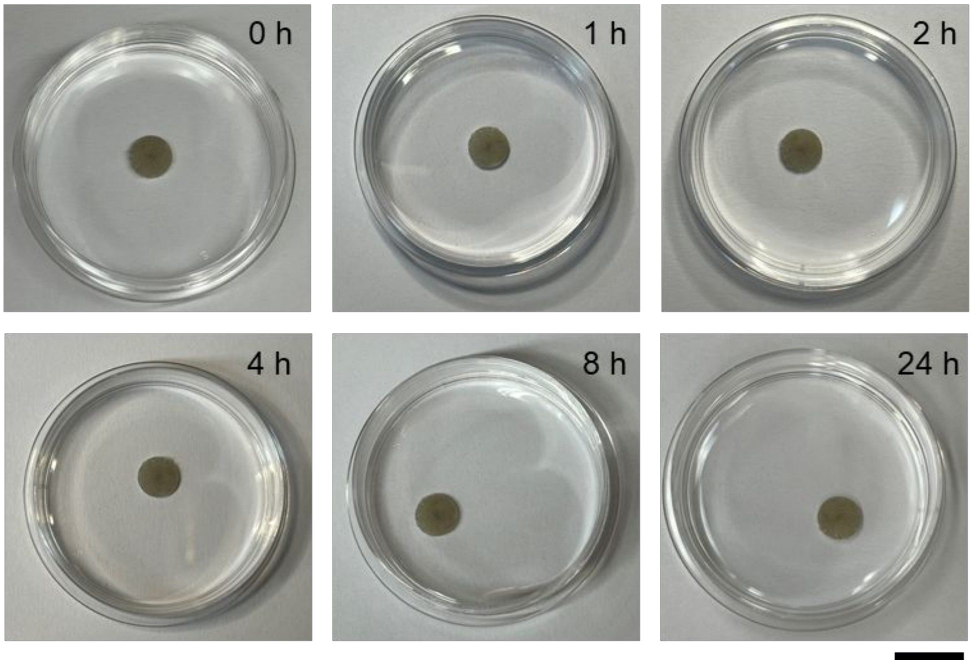

Figure S13. Optical images of the plasmonic biochip in artificial tears at different time points show the stability of the plasmonic biochip after 24 h of incubation in artificial tears. Scale bar 1 cm.

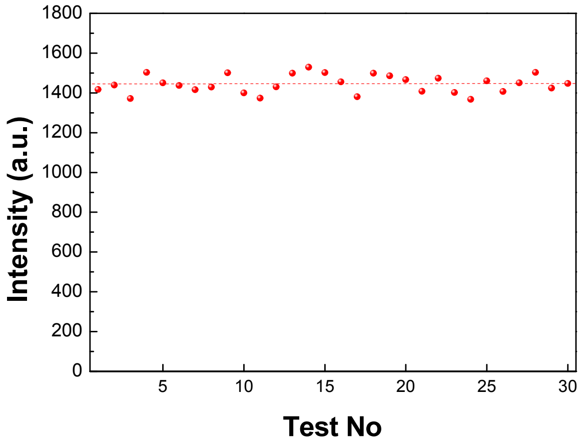

Figure S14. Raman intensities from 30 different regions across the plasmonic biochip with uric acid (200 μM) exposure show the reproducibility of the SERS substrate.

Enhancement factor (EF):

The enhancement factor (EF) of yolk-shell nanomaterials-decorated ZnO nanocages was calculated by using the following equation[3,4,5]:

$$\mathrm{EF} = \frac{I_{SERS}/N_{SERS}}{I_{bulk}/N_{bulk}} \qquad \text{S1}$$

Where I_{SERS} and I_{bulk} are intensities of the same band for the SERS and bulk spectra, respectively. N_{SERS} and N_{bulk} are the number of the 2-Naphthalenethiol (2-NT) molecules for the SERS and bulk spectra, respectively.

For yolk-shell nanomaterials with size of 59.9 nm in length and 30.2 in width, the volume of yolk-shell nanomaterial with AuNR core (53.5 nm in length and 14.5 nm in diameter) is estimated to be around $45800\ nm^3$.

I_{SERS} =~11500 counts and I_{bulk}=~350 counts

Raman spectrum of 2-NT in bulk was collected using 50x microscopy objective (with a numerical aperture (NA) =0.45). The approximate laser spot size of 50X objective can be obtained using the following expression:

$$\omega_0 = {0.61\lambda}/{\mathrm{NA}} \qquad \text{S2}$$

Where ω_0 is the minimum waist diameter for a laser beam of a wavelength λ focused by an objective with a numerical aperture NA.

$$\omega_0 = \frac{(0.61)(0.632)}{(0.45)} = 0.857\ \mu m \qquad \text{S3}$$

$$z_0 = \frac{2\pi\omega_0^2}{\lambda} = \frac{2\pi(0.857)^2}{0.632} = 7.302\ \mu m \qquad \text{S4}$$

The focal volume (τ) = $\left(\frac{\pi}{2}\right)^{1.5} \omega_0{}^2 z_0 = \left(\frac{\pi}{2}\right)^{1.5} \times 0.857^2 \times 7.302 = 10.558\ \mu m^3$ S5

Density of the 2-NT is 1.22 g/cm[3], molecular weight of 2-NT is 160.24 g/mol

$$N_{bulk} = \frac{(1.22)(10.558\ )(10^{-4})^3}{160.24} = 8.038 \times 10^{-14}\ \mathrm{mol} \qquad \text{S6}$$

$$N_{SERS} = \frac{(1.22)(45800)(10^{-7})^3}{160.24} = 3.487 \times 10^{-19}\ \mathrm{mol} \qquad \text{S7}$$

$$\mathrm{EF} = \frac{I_{SERS}/N_{SERS}}{I_{bulk}/N_{bulk}} = \frac{11500/3.487 \times 10^{-19}}{350/8.038 \times 10^{-14}} = 7.6 \times 10^6 \qquad \text{S8}$$

References

1. X. Huang, S. Neretina and M. A. El-Sayed, *Advanced Materials*, 2009, **21**, 4880-4910.
2. C. J. Orendorff and C. J. Murphy, *The Journal of Physical Chemistry B*, 2006, **110**, 3990-3994.
3. P. L. Stiles, J. A. Dieringer, N. C. Shah and R. P. Van Duyne, *Annual Review of Analytical Chemistry*, 2008, **1**, 601-626.
4. T. Itoh, M. Iga, H. Tamaru, K.-i. Yoshida, V. Biju and M. Ishikawa, *The Journal of Chemical Physics*, 2012, **136**, 024703.
5. L. Tian, N. Gandra and S. Singamaneni, *ACS Nano*, 2013, **7**, 4252-4260.
